# *In vitro* combinations of inert phenolato Ti(iv) complexes with clinically employed anticancer chemotherapy: synergy with oxaliplatin on colon cells†
†Electronic supplementary information (ESI) available. See DOI: 10.1039/c8ra00229k


**DOI:** 10.1039/c8ra00229k

**Published:** 2018-02-06

**Authors:** N. Ganot, E. Y. Tshuva

**Affiliations:** a The Institute of Chemistry, The Hebrew University of Jerusalem, Jerusalem 9190401, Israel. Email: edit.tshuva@mail.huji.ac.il

## Abstract

Advanced anticancer phenolato titanium(iv) complexes were combined with known chemotherapeutic anticancer drugs applied in the clinic and were analyzed *in vitro* on cell lines most sensitive to the Ti(iv) complex and relevant to the clinical application of the known drugs. Combination of the Ti(iv) complex with cisplatin on ovarian cells showed mostly an additive behavior, also on a line resistant to cisplatin. Combination of the Ti(iv) complex with fluorouracil on colon cells gave near additive behavior, and that with oxaliplatin gave a synergistic behavior at a wide range of Ti : Pt ratios, but only when the drugs were administered together. Increasing the time intervals between the administration of Ti and of Pt turned the behavior to antagonistic, suggesting some deactivation of Pt by the Ti agent. For combinations where the drugs were applied together, the behavior depended on the effect level, and higher effects gave greater synergism, implying that technical aspects such as solubility are influential. Nevertheless, more complex patterns recorded for combinations where the drugs had been applied separately suggested multiple mechanisms with different concentration dependence. Overall the results point to high medicinal potential for the tested compounds for anticancer combination treatments.

## Abstract 2

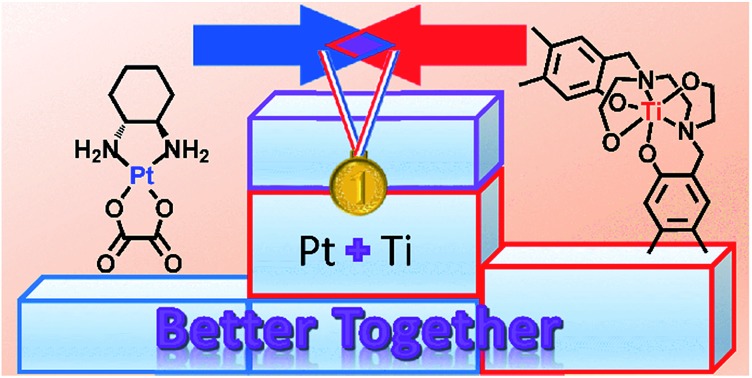
An advanced phenolato Ti(iv) complex combined with known cancer drugs on the clinically-relevant lines provides synergistic or additive *in vitro* anticancer activity.

## Introduction

**Chart 1:**
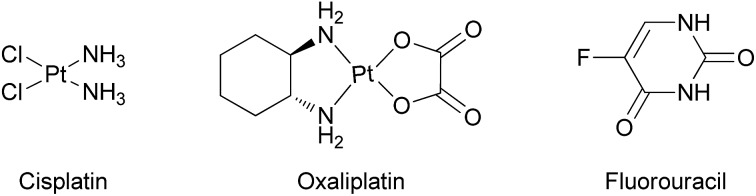
Cisplatin, oxaliplatin, and fluorouracil.

**Chart 2:**
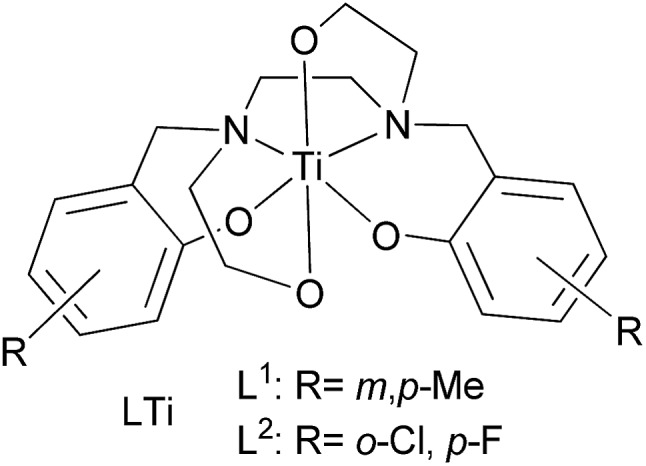
Diaminobis(phenolato)-bis(alkoxo)Ti(iv) complexes.

Cisplatin (Chart 1) and its derivatives are significant components in the chemotherapeutic treatment of many types of cancer.^1^–^3^ Cisplatin is often used for ovarian, testicular, and bladder cancers, applied mostly in combination with other drugs.^4^–^12^ Oxaliplatin (Chart 1), a second generation platinum-based drug, serves as a common treatment for colon cancer, especially in combination with the thymidylate synthase inhibitor fluorouracil (Chart 1).^13^–^15^ Nevertheless, the high toxicity of the platinum ion and the development of drug resistance in many cases encourage scientists to search for other metal based drugs.^16^–^24^ Among the metals studied, titanium based complexes exhibit reduced toxicity and wide activity range, without resistance development known to date.^25^–^37^ In particular, the advanced diaminobis(phenolato)-bis(alkoxo)Ti(iv) complexes that we have introduced show enhanced cytotoxic activity and exceptional water resistance with no decomposition for weeks in water solutions and no activity decrease following one week in biological medium.^38^ Importantly, the leading phenolato Ti(iv) complex L^1^Ti (Chart 2) not only showed efficacy *in vivo* in reducing mortality in treated mice inoculated with lymphoma, but also showed no clinical signs of toxicity to the treated animals.^38^ This complex also exhibited promising results when evaluated on the NCI-60 panel of the Developmental Therapeutics Program (DTP) of the National Cancer Institute (NCI), with a wide range of activity and significant cytotoxicity toward all cell lines tested, whereby especially high sensitivity was recorded for colon and ovarian cell lines.^38^

Combination therapy is therefore a common method for treating cancer diseases. Combination of two or more drugs enables achieving the desired effect but with reduced dose of each drug, which may consequently reduce the side effects of the drugs and the chances for resistance development.^39^,^40^ In a previous research, early generation of phenolato Ti(iv) (“salan”) complexes displayed synergistic and additive behaviours when combined with randomly selected organic or inorganic anticancer drugs for *in vitro* testing on certain cell types.^41^ Herein, specific *in vitro* combinations of the advanced phenolato Ti(iv) complexes with known drugs are presented; the NCI-60 results provide an opportunity to select the most suitable cell lines for analysis. Thus, the lines were chosen as those particularly sensitive to L^1^Ti based on the NCI-60 screen, namely, ovarian and colon lines,^38^ and the combined drugs were chosen as those commonly employed in the clinic for a tested cell type, namely, cisplatin and oxaliplatin/fluorouracil, respectively. Additive and synergistic behaviours were often detected, both with a significant medical value.

## Experimental

The ligands L^1^H_4_ and L^2^H_4_ and their complexes L^1^Ti and L^2^Ti were synthesized as previously described.^38^ *cis*-Dichlorodiammine platinum(ii) 99% was purchased from Acros, 5-fluorouracil 99% was purchased from Apollo Scientific Ltd, and oxaliplatin was purchased from Glentham Life Sciences Ltd.

Cytotoxicity was measured on HT-29 colon cancer cells obtained from ATCC Inc, and A2780 ovarian and A2780-cp cisplatin-resistant ovarian cancer cells obtained from ECACC Inc., using the MTT assay as previously described.^42^ Approximately 0.6 × 10^6^ cells in medium (contains: 1% penicillin/streptomycin antibiotics; 1% l-glutamine; 10% fetal bovine serum (FBS) and 88% medium RPMI-1640, all purchased from Biological Industries Inc.) were seeded into a 96-well plate and allowed to attach for a day. The cells were consequently treated with the reagent or combination of reagents tested at fixed ratios at 10 different concentrations. After a standard of 3 days incubation at 37 °C in 5% CO_2_ atmosphere, MTT (0.1 mg in 20 μL) was added and the cells were incubated for additional 3 hours. For experiments where the Pt complex was inserted with certain time intervals after the Ti complex, the incubation time was measured starting from the first administration. After the incubation period, the MTT solution was removed, and the cells were dissolved in 200 μL isopropanol. The absorbance at 550 nm was measured by a Bio-Tek EL-800 microplate reader spectrophotometer or by a Spark 10M Multimode Microplate Reader spectrophotometer. Relative IC_50_ values were determined by a nonlinear regression of a variable slope (four parameters) model by Graph Pad Prism 5.04 program, with error values based on the STD of the at least 3 × 3 repetitions (three separate measurements conducted on three different days to give nine repeats altogether). Some dose response curves can be found in the ESI.†

The interactions between the combined reagents were evaluated with the isobolographic method and the multiple effect analysis (based on the median effect principle),^43^,^44^ using Graph Pad Prism 5.04 program.

Pt cellular accumulation studies were conducted using ICP-MS Agilent 7500cx (Agilent Technologies Inc., USA). HT-29 cells were cultured in 6-well plates at density of ∼800 000 cells per well and allowed to attach overnight. Cisplatin, oxaliplatin, or the combination of each with L^1^Ti complex were separately added for incubation of 24 h (37 °C, 5% CO_2_ atmosphere). The medium was then removed and the cells were washed three times with DPBS (purchased from Biological Industries Inc., Israel). The cells underwent three freeze/thaw cycles, and total protein was determined using the Lowry protein assay.^45^,^46^ A known amount of each sample was lyophilized using a VirTis Benchtop K Lyophilized (SP industries, USA). The dried cell samples were dissolved in >68% HNO_3_ (Primar Plus – Trace analysis grade, purchased from Fisher Chemical) and were left overnight for complete cell digestion and degradation under heat. The samples were then dissolved in 1% HNO_3_ in TDW (obtained from PURELAB Classic, ELGA). The Pt concentration in the cell samples are presented as a mean ± SD of three independent experiments relative to the control (Pt complex alone) normalized to 100%.

## Results and discussion

### General

Two phenolato Ti(iv) complexes, L^1^Ti and L^2^Ti (Chart 2), were synthesized as previously published, as confirmed by ^1^H NMR.^38^ The cytotoxicity of these complexes alone and in combination with known drugs – cisplatin, oxaliplatin, and fluorouracil, was measured by the MTT assay according to a published protocol (See ESI† for dose–response curves).^42^ The behaviours of the combinations were first analysed by the well-established isobolographic method.^43^,^44^ An isobologram graph represents the concentrations of the drugs required to achieve a defined effect, normally applied for inhibition of 50% of cell viability. Each axe presents the concentration of one of the combined drugs; the IC_50_ values of each drug when administrated alone are plotted on the axes, and the line connecting these dots is the additive line, which represents eqn (1) when the combination index (CI) is 1 ((IC_*x*_)_A,B_: the concentration of drug A and B that inhibits *x*% of cell viability when administrated alone; *C*_A_, *C*_B_: the concentration of drugs A, B that inhibits *x*% of cell viability when administrated in combination).1
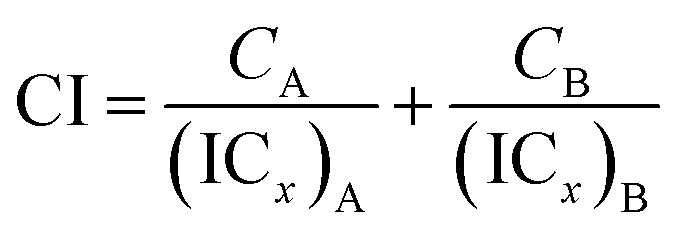



The additive line is accompanied by the error range presented by dashed lines, as derived from the error values of the individual IC_50_ values. In the combination experiment the drugs are combined at a fixed ratio, the 50% cell growth inhibition point is identified, and the concentration of each drug at that point is plotted on the isobologram graph. Dots located above the additive region indicate antagonistic behaviour, and those located bellow it indicate synergism.

### Combinations on ovarian cancer cells

**Fig. 1:**
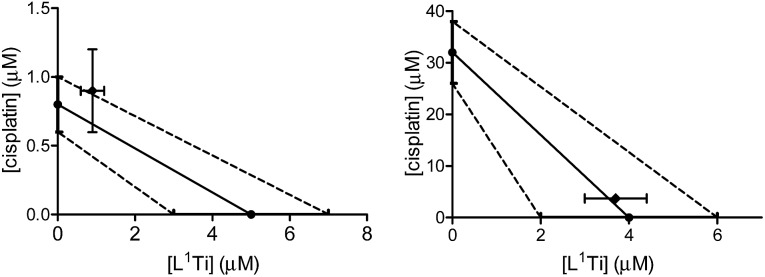
Isobolographic analysis of the anti-proliferative activity of the 1 : 1 combination of L^1^Ti with cisplatin against human ovarian A2780 (left) and cisplatin-resistant human ovarian A2780-cp (right) cell lines.

L^1^Ti was combined with cisplatin, commonly employed in the clinic for ovarian cancer,^8^,^9^ and the combination was tested on human ovarian A2780 cell line and cisplatin-resistant human ovarian A2780-cp cell line. The drugs were combined at a fixed 1 : 1 ratio. The results are presented in Fig. 1.

The combination on both cell lines showed additive or near-additive behaviours. This implies that the two anti cancer agents act as if they were administered alone, and there are no significant beneficial or destructive interactions between them. The even more profound additive effect on the line resistant to cisplatin implies that the presence of the Ti complex did not affect the cell resistance,^38^,^47^–^49^ to the better or to the worse. Nevertheless, the additive behaviour has a marked medicinal value, as it should enable lowering the dose of the toxic cisplatin.

### Combinations on colon cancer cells

**Fig. 2:**
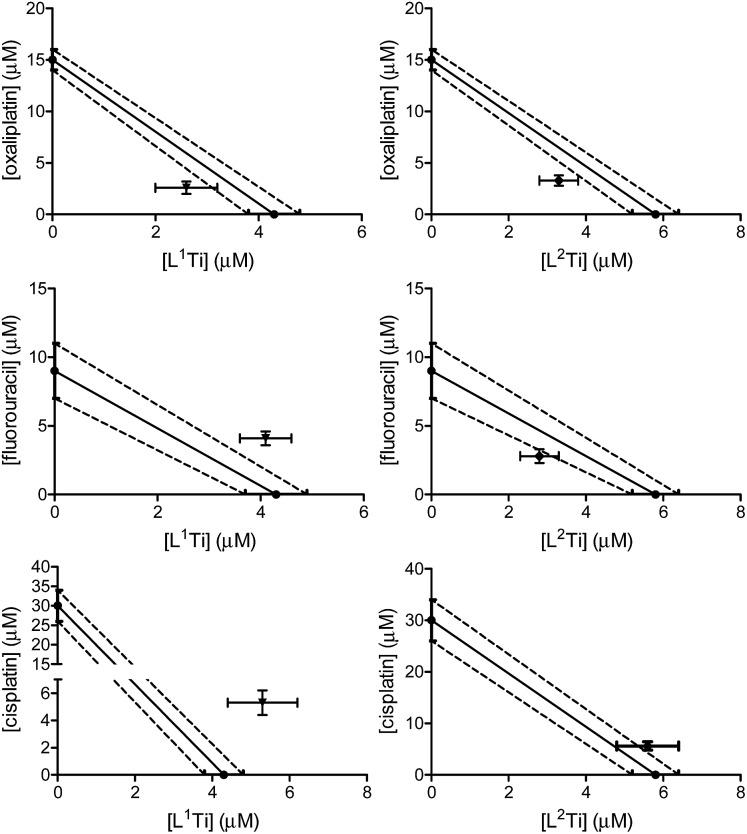
Isobolographic analysis of the anti-proliferative activity of the 1 : 1 combinations of L^1^Ti (left) and L^2^Ti (right) complexes separately with oxaliplatin (top), fluorouracil (middle), and cisplatin (bottom), against human colon HT-29 cell line.

The cytotoxicity of L^1,2^Ti, separately combined with the colon cancer drugs oxaliplatin and fluorouracil,^13^–^15^ was tested on human colon HT-29 cells. Cisplatin was also evaluated as a lead drug for comparison. The drugs were first combined at a fixed 1 : 1 ratio. The results are presented in Fig. 2.

The combinations of both complexes with oxaliplatin showed a synergistic behaviour. This behaviour may result from beneficial interactions between the drugs, related mechanisms of action, or simply improved technical aspects such as solubility or cellular penetration. The combinations of fluorouracil with L^1,2^Ti exhibited near additive behaviours, where that of L^1^Ti is more antagonistic and that of L^2^Ti is on the borderline with synergistic behaviour. Because these complexes are suspected to operate by a similar (although still undetermined) mechanism, it is yet to be concluded whether this difference is indeed meaningful, as different ligand substitution was shown previously to impact the behaviours of the combination.^41^ Nevertheless, it is not unlikely that these differences simply derive from different solubility/accessibility of the differently substituted complexes.

Interestingly, combinations of both complexes with cisplatin on the colon cancer cells showed an antagonistic or near-antagonistic behaviour. Because cisplatin is not a common drug to treat colon cancer and provides relatively high IC_50_ values,^50^,^51^ it is reasonable that the big difference in IC_50_ values between the two compounds would result in decreased accuracy of the isobolographic analysis. Nevertheless, some destructive interaction between the drugs cannot be ruled out.

**Fig. 3:**
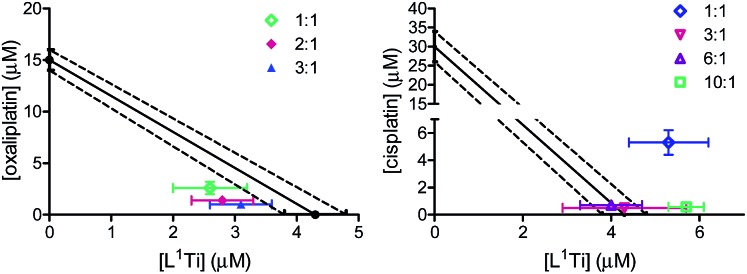
Isobolographic analysis of the anti-proliferative activity of the combinations of L^1^Ti with oxaliplatin (left) and cisplatin (right) at different ratios against human colon HT-29 cancer cells.

One most promising combinations showing a synergistic behaviour, L^1^Ti with oxaliplatin, were selected for further studies. Because previous studies showed that the ratio of the combined agents may affect the behaviour of the combination,^41^ L^1^Ti was combined with oxaliplatin at different ratios up to 3 : 1, Ti : Pt (Fig. 3). All the ratios tested consistently showed synergistic behaviour, with little impact of the particular ratio on the behaviour of the combination. This observation provides additional flexibility to an envisioned potential treatment, especially as the Ti agent shows markedly reduced side effects.^38^,^47^,^52^–^54^ Interestingly, combinations of L^1^Ti with cisplatin at different rations analysed for comparison showed a clear impact of the ratio employed (Fig. 3). Although the 1 : 1 ratio gave antagonism, increasing the ratio in favour of Ti turned the behaviour to additive, whereby increasing further the ratio to 10 : 1 Ti : Pt turned the behaviour back to antagonistic. This may again derive from the potentially decreased accuracy of the analysis due to the low activity of cisplatin relative to that of L^1^Ti.

**Fig. 4:**
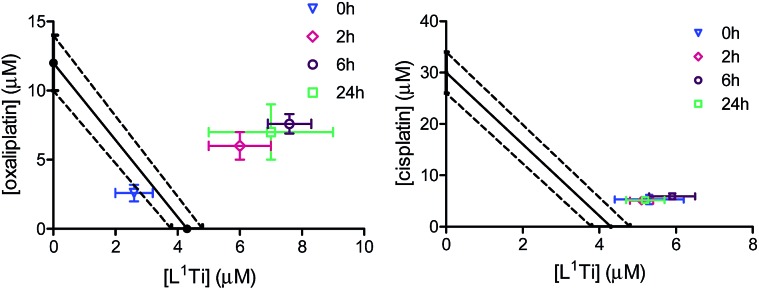
Isobolographic analysis of the anti-proliferative activity of the combinations of L^1^Ti with oxaliplatin (left) and cisplatin (right) against human colon HT-29 cancer cells with different time intervals between administrations.

To shed additional light on possible interactions between the combined drugs and their time dependence,^41^ the combinations of L^1^Ti with oxaliplatin or cisplatin in a 1 : 1 Ti : Pt ratio were analysed with varying time intervals between the administration of the two drugs, applying the Ti(iv) complex first (Fig. 4). Interestingly, similar IC_50_ values were obtained in all experiments involving combination with cisplatin, regardless of the period between administrations. This observation supports the notion that the antagonistic behaviour observed for the 1 : 1 ratio of the drugs applied together is not a result of specific destructive interactions, which should have been reduced with increasing the time between administrations. For oxaliplatin, however, the behaviour became antagonistic when the drugs are not applied simultaneously, as observed previously for related combinations.^41^ This behaviour may reflect either a beneficial interactions of the drugs upon mixing, or cellular interactions of L^1^Ti that hamper the reactivity of oxaliplatin or affect its cell entry.

**Fig. 5:**
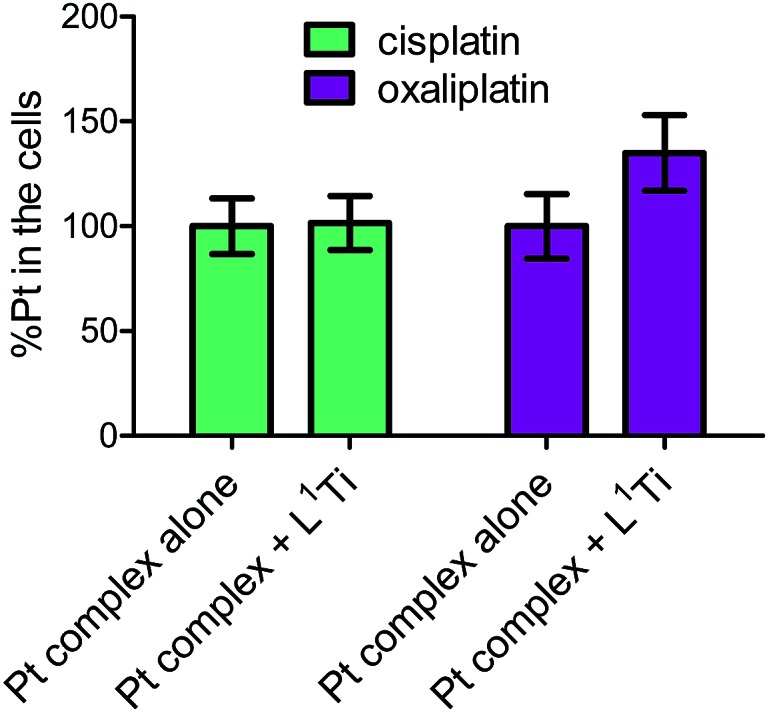
Normalized Pt levels in cells treated with cisplatin (90 μM) or oxaliplatin (36 μM) with or without L^1^Ti (13 μM).

To further examine whether L^1^Ti affects cell entry of cisplatin or oxaliplatin as a source of the antagonistic or synergistic behaviors, respectively, the amount of Pt in the cells after treatment with cisplatin/oxaliplatin alone, or combined with L^1^Ti, was determined. Colon HT-29 cells were exposure to the platinum complex at 3× IC_50_ concentrations (90 μM for cisplatin; 36 μM for oxaliplatin) or the platinum complex at the same concentration combined with L^1^Ti (3× IC_50_; 13 μM) for 24 hours. The total protein as a measure of the number of cells was determined by the Lowry protein assay, and the Pt concentration was measured by ICP-MS (Fig. 5). L^1^Ti did not impact Pt entry to cells, neither for cisplatin nor for oxaliplatin, with similar Pt concentrations obtained for both experiments. Similar results were obtained when the Ti was administered prior to Pt administration (see ESI†). These results rule out altered cell entry as a source for the antagonistic/synergistic behaviors.

### The multiple drug effect analysis

The isobologram analysis, although can conveniently present several experiments on a single plot, is limited to the presentation of only a single effect for each experiment (herein 50% cell growth inhibition). The combination index analysis of multiple effects (based on the median effect principle)^43^,^44^,^55^ presents the behaviour of a combination in a given experiment at various cell growth inhibition levels. The *x* axe represents the different effect levels, and the *y* axe is the calculated combination index (CI) according to eqn (1). CI = 1 represents an additive behaviour, CI < 1 represents synergism, and CI > 1 represents antagonism. This more complex analysis was therefore applied for selected experiments to get a deeper understanding of the parameters of influence.

**Fig. 6:**
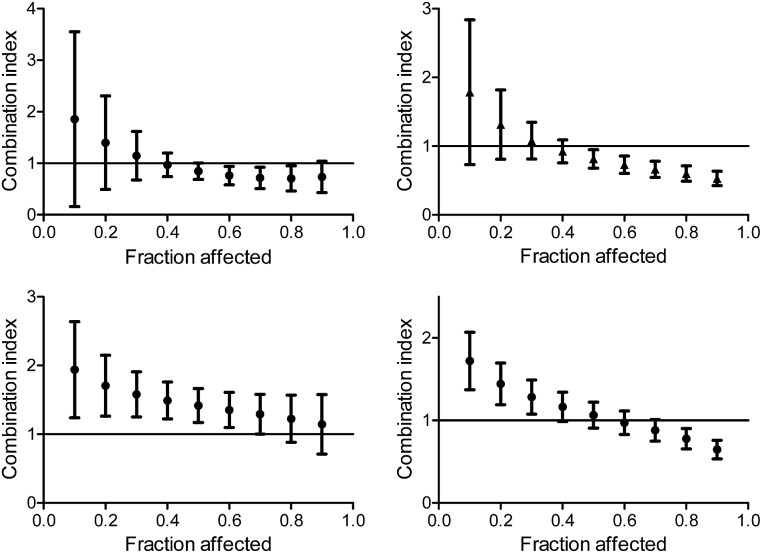
Combination index for different effects on human colon HT-29 cancer cells by the combination of L^1^Ti with oxaliplatin (top) and cisplatin (bottom) at ratios 1 : 1 (left) and 1 : 3 (right).

The CI curves of the combination of L^1^Ti with oxaliplatin or cisplatin at different ratios (Fig. 6, ESI†) interestingly demonstrate that the behavior of the combination is effect-dependent, and a behavior observed for the mid-point (50% cell growth inhibition) is not necessarily the same for all effects. The combinations studied herein for different ratios of the compounds administered together all exhibit a similar trend, where the larger the affect, the stronger the synergism (or the weaker the antagonism). Thus, aiming at maximal effects, this relatively wide synergism obtained for the combination with oxaliplatin is highly beneficial, as the antagonism at low effects is medically insignificant. Additionally, for the combination with cisplatin, some high effects in the 3 : 1 and 6 : 1 Ti : Pt ratios also fall in the near synergistic zone (Fig. 6, ESI†), although the edge ratio points tested of 1 : 1 and 10 : 1 combinations are completely antagonistic for all effects.

**Fig. 7:**
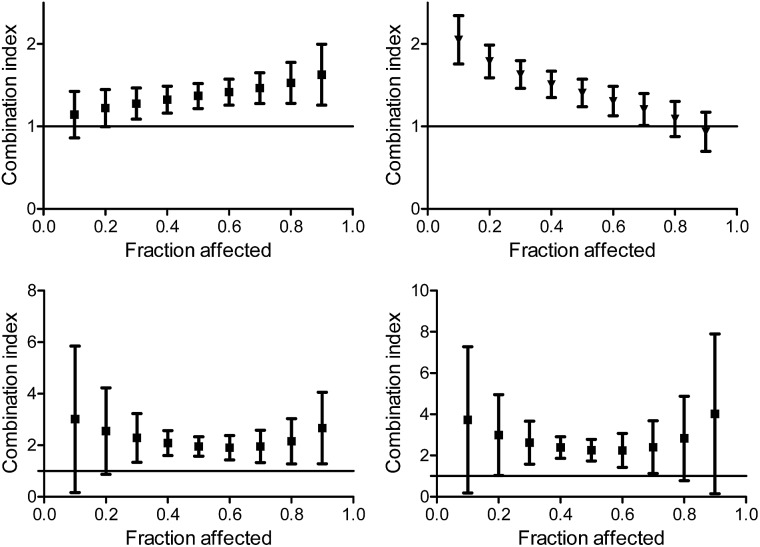
Combination index for different effects on human colon HT-29 cancer cells by the combination of L^1^Ti with cisplatin (top) and oxaliplatin (bottom) with 2 h (left) and 24 h (right) between the administration of the agents.

When applying the multiple effect analysis on the measurements with time intervals between administrations, the behavior remained antagonistic for all effects but a different trend is observed (Fig. 7); whereas for the combination with oxaliplatin, the mid effects are least antagonistic, for the combination with cisplatin the trend depends on the period between administrations. This complicated behavior may reflect more than a single mechanism ongoing for each drug, with different concentration dependence.

## Conclusions

This paper presents the *in vitro* combinations of two advanced phenolato Ti(iv) complexes with known, clinically employed anticancer chemotherapeutic drugs, on lines that were identified as particularly sensitive to the Ti(iv) agent based on the NCI-60 screen. When the combined drugs were analysed on the lines relevant to their reactivity in the clinic, mostly additive and synergistic behaviours were observed; specifically, combination with cisplatin on ovarian cells gave an additive behaviour, and combination with oxaliplatin on colon cells gave synergism at various ratios. Both behaviours are medically valuable to potentially enable lowering the doses of each drug to reduce side effects, especially that of the more toxic platinum drug.

The Ti(iv) complex did not affect the penetration of the Pt complexes to the cancer cells; nevertheless, it is important to administer the combined drugs simultaneously to achieve the optimal effect.^41^ It thus cannot be ruled out that activation of some cellular pathways by the Ti(iv) complex when it is administered prior to the Pt agent may hamper the reactivity of the Pt compound, developing partial Pt resistance. This is especially plausible as the more profound CI analysis of multiple effect levels clearly indicated antagonism for all effect points when the compounds were administered with certain time intervals in between, although different patterns of concentration dependence were detected; this implies that multiple mechanisms with different concentration dependence may be ongoing for one or both of the drugs. Interestingly, this analysis run on simultaneously applied combinations often showed behaviour change upon concentration increase, implying that the reasons for non-additive behaviours in these cases more likely relate to technical aspects (solubility, accessibility), rather than complex cellular bio-interactions. It is thus a plausible conclusion that different mechanisms are ongoing for the two drugs, especially concerning the COMPARE analysis previously reported, showing no distinct correlations of the results obtained on the NCI-60 panel with those of known drugs.^38^ It is yet to be determined whether the target of the Ti(iv) complexes is also DNA.^36^

To conclude, we found that specific combinations of promising anticancer phenolato Ti(iv) complexes with known drugs applied simultaneously at a range of possible ratios on the relevant cancer cells were mostly additive or synergistic. The behaviour may change depending on the effect level, and mostly, the higher the effect level the better is the behaviour, as would be preferred for medicinal applications. The results presented herein call for additional carefully designed and clinically relevant *in vivo* combination studies, which are currently ongoing in our laboratory.

## Conflicts of interest

There are no conflicts to declare.

## Supplementary Material

Supplementary information
