# Supplementary material for: In vitro combinations of inert phenolato Ti(iv) complexes with clinically employed anticancer chemotherapy: synergy with oxaliplatin on colon cells
Source: RSC Adv. 2018 Feb 6;8(11):5822–7. doi: 10.1039/c8ra00229k (PMC6003540; doi:10.1039/c8ra00229k)
Supplement: RA-008-C8RA00229K-s001 [file RA-008-C8RA00229K-s001.pdf]

Electronic Supplementary Material (ESI) for RSC Advances.
This journal is © The Royal Society of Chemistry 2018

In vitro Combinations of Inert Phenolato Ti(IV) complexes with Clinically Employed Anticancer Chemotherapy: Synergy with Oxaliplatin on Colon Cells

Electronic Supplementary Information

Nitzan Ganot, Edit Y. Tshuva*

The Institute of Chemistry,

The Hebrew University of Jerusalem,

Jerusalem 9190401 Israel.

Email: edit.tshuva@mail.huji.ac.il

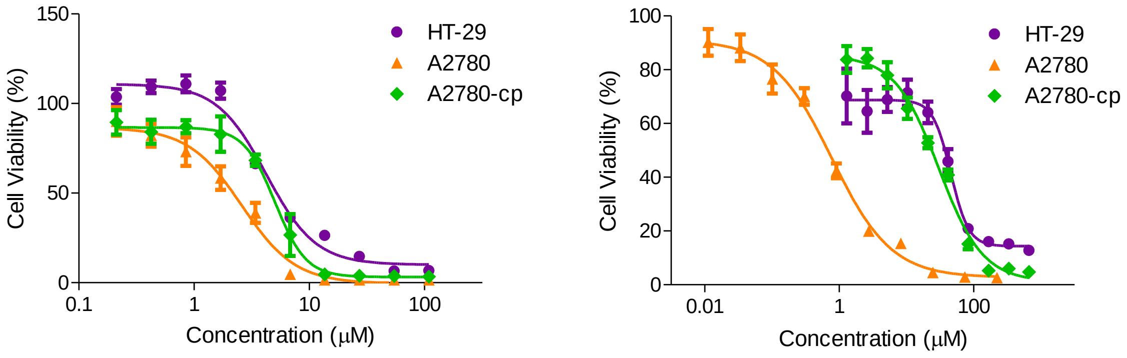

Fig. 1. Dose-response curves of L[1]Ti (left) and cisplatin (right) against human colon HT-29 (IC_{50} = 4.3 ± 0.5 and 30 ± 4 μM, respectively), human ovarian A2780 (IC_{50} = 5 ± 2 and 0.8 ± 0.2 μM, respectively), and cisplatin-resistant human ovarian A2780-cp (IC_{50} = 4 ± 2 and 32 ± 6 μM, respectively) cell lines (including 3-day incubation period; based on 3 times 3 repetitions).

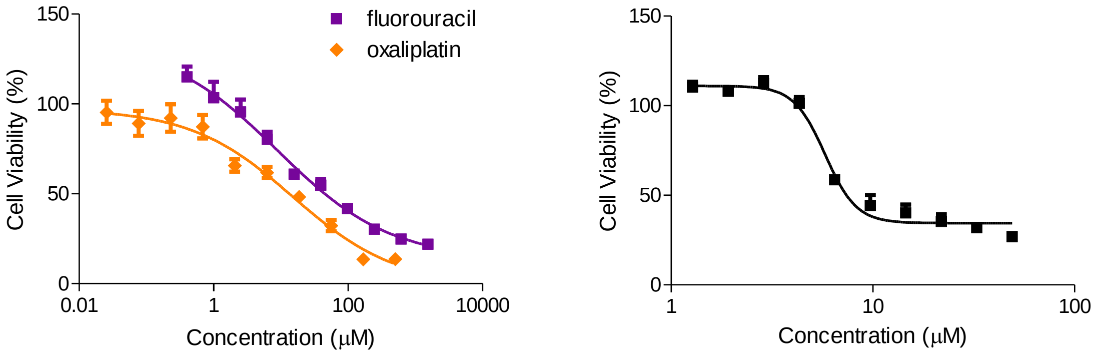

Fig. 2. Dose-response curves of fluoruracil and oxaliplatin (left) and L[2]Ti (right) against human colon HT-29 cell lines (IC_{50} = 9 ± 2, 12 ± 2 and 5.8 ±0.6 μM, respectively). (including 3-day incubation period; based on 3 times 3 repetitions).

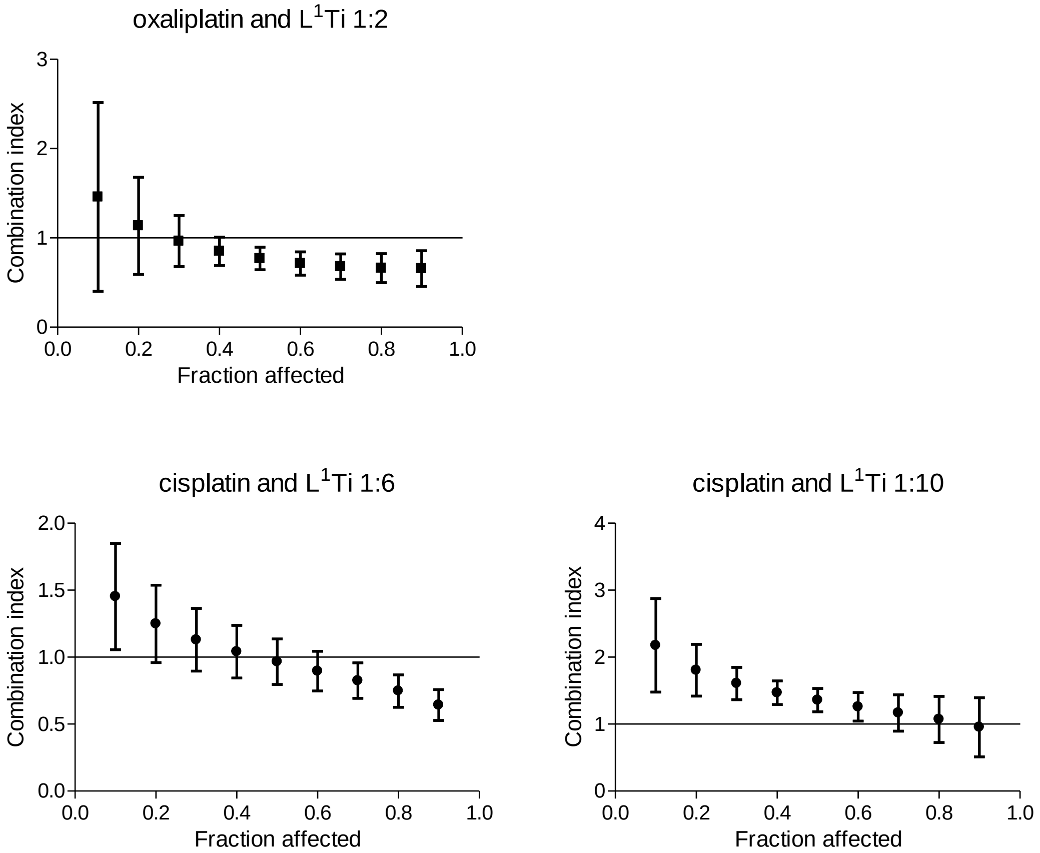

Fig. 3. Combination index for different effects on human colon HT-29 cancer cells by the combination of L[1]Ti with oxalipltin (top) and cisplatin (bottom) at different ratios

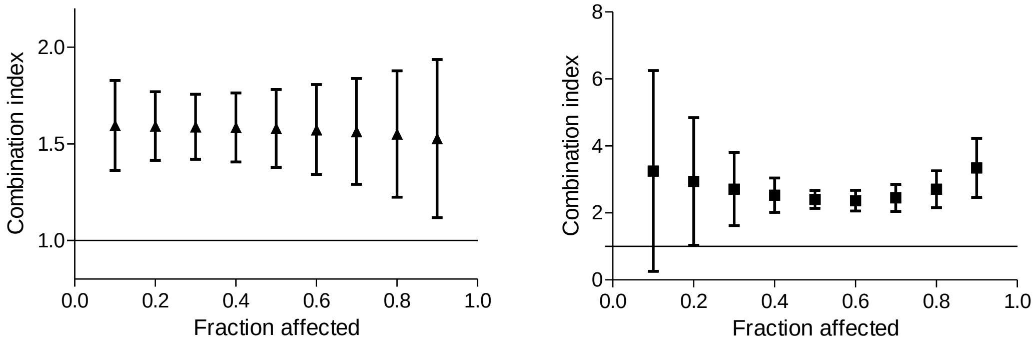

Fig. 4. Combination index for different effects on human colon HT-29 cancer cells by the combination of L[1]Ti with cisplatin (left) and oxaliplatin (right) at time interval of 6 hours between the administration of the agents.

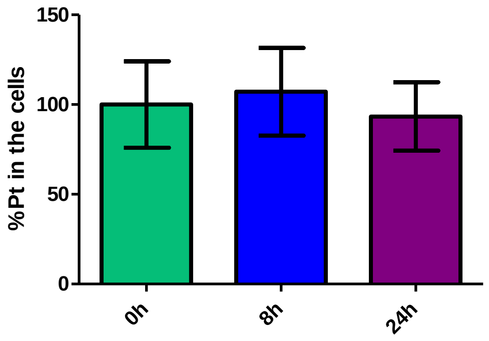

Fig. 5. Pt levels in cells treated first with L^{1}Ti (8.6 µM) and after varying time interval with cisplatin (60 µM); the overall incubation time was 48 hours.

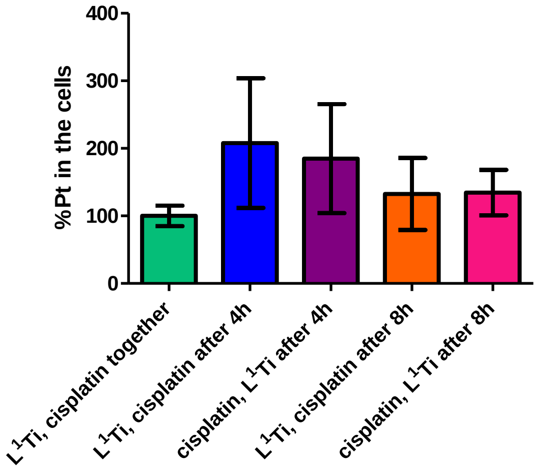

Fig. 6. Pt levels in cells treated with L^{1}Ti (8.6 µM) and cisplatin (60 µM) at varying time interval between the administration of the agents; the overall incubation time was 24 hours.
